# The effect of the preferred hand on drawing movement

**DOI:** 10.1038/s41598-023-34861-x

**Published:** 2023-05-22

**Authors:** Zinat Zarandi, Natale Adolfo Stucchi, Luciano Fadiga, Thierry Pozzo

**Affiliations:** 1 IIT@UniFe Center for Translational Neurophysiology of Speech and Communication, Istituto Italiano di Tecnologia, via Fossato di Mortara 17/19, 44121 Ferrara, Italy; 2 Department of Psychology, University of Milano-Bicocca, Milan, Italy; 3 IIT@UniFe Center for Translational Neurophysiology, Section of Human Physiology, Istituto Italiano di Tecnologia, University of Ferrara, Ferrara, Italy; 4 INSERM UMR1093-CAPS, UFR des Sciences du Sport, Université Bourgogne Franche-Comté, Dijon, France

**Keywords:** Neuroscience, Cognitive neuroscience

## Abstract

The observation that different effectors can execute the same movement suggests functional equivalences driven by limb independent representation of action in the central nervous system. A common invariant motor behavior is the speed and curvature coupling (the 1/3 power law), a low dimensional (abstract) descriptor of movement which is resilient to different sensorimotor contexts. Our purpose is to verify the consistency of such motor equivalence during a drawing task, by testing the effect of manual dominance and movement speed on motor performance. We hypothesize that abstract kinematic variables are not the most resistant to speed or limb effector changes. The results show specific effects of speed and hand side on the drawing task. Movement duration, speed-curvature covariation, and maximum velocity were not significantly affected by hand side, while geometrical features were strongly speed and limb dependent. However, intra-trial analysis performed over the successive drawing movements reveals a significant hand side effect on the variability of movement vigor and velocity-curvature relationship (the 1/3 PL). The identified effects of speed and hand dominance on the kinematic parameters suggest different neural strategies, in a pattern that does not go from the most abstract to the least abstract component, as proposed by the traditional hierarchical organization of the motor plan.

## Introduction

The presence of kinematic regularities in human movements performed in different sensorimotor contexts suggest a limited number of motor solutions. Motor invariants as generic behavioral descriptors echo the idea of motor equivalence, a term initially referring to the fact that one finds alternative solutions for achieving the same goal^1–6^. In theory, this assumes that different effectors can execute the same action planned in cortical areas independent of the primary representation of the effector. In other words, the motor representations of the secondary areas are functional and abstract (abstract here refers to a low dimensional representation) by contrast to a one-to-one somatotopic mapping where motor cortex neurons represent muscle activity. Following such a framework, the movement features being constant, with respect to any end effector, would be controlled by the top level of a hierarchical system. At a lower level, metrical characteristics (e.g. muscle force in amplitude or duration) would allow the stable expression of the task in various sensorimotor contexts^2^.

Writing tasks, characterized by a consistent relationship between time and space descriptors of hand movement, illustrate the functional equivalence principle. To its end, a systematic covariation between movement curvature and velocity, namely the 1/3 Power Law (1/3 PL)^7^, explained by the speed of movement is proportional to the − 1/3 power of the radius of curvature (or − 2/3 power of the local curvature). This phenomenon has been observed during transitive tasks (e.g. arm reaching and locomotion), and intransitive tasks (e.g. drawing an ellipse) performed with different end effectors (e.g. hand, foot, eye, and lip movements)^8^. Further supporting the hypothesis of an abstract representation of action and the concept of motor equivalence, other studies show that imagined movements also conform to the 1/3 PL^9,10^.

On the basis of the above-mentioned results, action would thus be planned according to a top-down scheme, from executive functions^11^controlling a cascade of operations going from preparatory planning, then to the translation of the motor plan into limb trajectory, proceeding into kinematic variables, and finally expressed through muscle activity^12^. However, other evidence showed that peripheral constraints (e.g. the visco-elastic nature of the mechanical plant that implements the commands) could nevertheless affect the expression of motor invariant^13,14^. In addition, speed-curvature covariation has been observed during larvae of Drosophila locomotion^15^, indicating that low-level executive neural systems can generate trajectories that follow the so-called 1/3 PL. Thus, low dimensional representations of action might not exclusively follow a top-down organization but may rely on distributed neural networks involving multiple cortical and subcortical structures. From these empirical observations, the notion of motor equivalence, which supposes a hierarchical stratification of motor representations, raises several interesting questions: when multiple factors constrain a task, what motor regularities subsist? For example, consider one low-dimensional kinematic parameter inside a gradual motor planning organization: when one acts under unusual conditions (e.g. writing or drawing with the non-dominant hand), will this parameter be affected less than others supposedly linked to somatotopic representation and, therefore, more sensitive to effector selection?

Surprisingly, while the motor equivalence of speed-curvature covariation has been investigated when changing the amplitude^16^ or the type of body motion^17–19^, the ability to draw with either hand with equal proficiency is scarcely documented. However, the study of motor performance of limbs with the same musculoskeletal system allows the exclusion of potential artifacts due to local factors. Thus, these inter manual differences can only result from differentiated motor commands rather than from articular, muscular, or dynamic anatomical properties. Similarly, motor invariants resulting from motor commands directed to homologous muscle groups could reveal movement parameters shared across both hemispheres. For example, there is currently no evidence demonstrating that the locus of motor equivalence is in the dominant hemisphere to the best of our knowledge.

By using the potentially disruptive effects of a task performed with the non-dominant (ND) hand at different speeds, we aim to extract motor characteristics that resist these disturbances. We hypothesize that (a) the dimensionality of the variables does not predict their resistance to velocity or limb effector changes and (b) that the so-called abstract variables may be affected during execution by the ND hand with respect to the limb-dependent kinematic parameters that implement the task on different end effectors.

**Figure 1:**
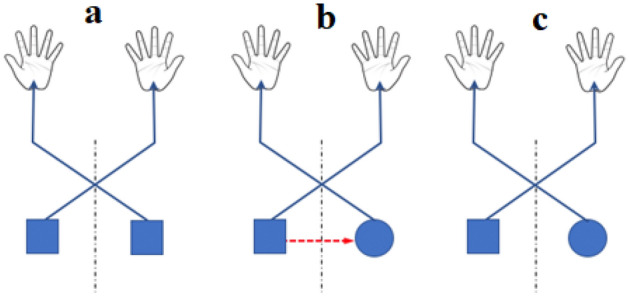
Schema illustrating the framework hypotheses: three idealized block diagrams modeling the motor command of the drawing movement performed with the dominant (right hand) or the non-dominant hand (left hand). Square and circle represent functional modules projecting downstream towards upper limb muscles (continuous blue arrows upwards). The dashed midline shows interhemispheric separation. (**a**) Motor command originating from a generic functional motor equivalence, non-lateralized, end effector independent, resulting in bilateral activation of the right and left homologous muscle groups. (**b**) The motor command is now lateralized in the dominant hemisphere (left blue square) and as suggested by the red dashed horizontal dotted line, the motor information generated in the dominant hemisphere transmits through a crossed corticospinal tract to a functional module in the non-dominant hemisphere (right blue circle). (**c**) The drawing movement results from asymmetric motor commands originating from different hand drawing functional modules in the last option.

More precisely, Fig. 1 summarizes our framework and proposes three hypothetical mechanisms of the drawing task performed with the D (dominant) and the ND hand. Following the first mechanism, the movement could result from a motor equivalence (end effector-independent), non-lateralized, projecting the same signal on the two modules (e.g. on the primary motor cortices, in blue squares) driving the right and left homologous muscle groups (Fig. 1a). This option (non-lateralized motor output) predicts a similar motor performance on both hands for which both hemispheres contain the same information. In the second mechanism, ND hand movement could originate from a lateralized module in the dominant hemisphere. In this case, the motor details transmit through callosal pathways to a module located in the ND hemisphere (lateralized motor output, Fig. 1b). This diagram which assumes a hemispherical asymmetry, predicts a partial degradation of the ND hand performance due to the implementation of the drawing task undergone with the ND hand. In the last mechanism, the motor performance could result from asymmetric modules (left and right blue squares, Fig. 1c). A strict dependency of movement features on the manual dominance, validated by a significant effect of handedness on the kinematic variables, would confirm such a diagram. To this aim, we consider both performance accuracy and precision to determine the robustness levels of each movement component when executed in unusual conditions.

## Results

Table 1 lists the descriptive data expressed as mean and standard deviation (SD) of all parameters across 40th subjects. The extracted parameters analyzed in three main categories included: kinematic, geometrical, and 1/3 power law parameters.

**Table 1:** Mean and standard deviation of kinematic and geometrical variables.

	Mean						SD					
	ND-S	ND-N	ND-F	D-S	D-N	D-F	ND-S	ND-N	ND-F	D-S	D-N	D-F
MaxV (cm/s)	14.733	29.235	44.302	15.123	28.965	44.842	5.880	6.156	7.255	6.005	6.699	6.767
Mean V (cm/s)	9.701	19.307	28.896	9.888	19.080	29.316	3.759	4.066	4.678	3.921	4.351	4.410
Duration (s)	3.656	1.663	1.082	3.629	1.678	1.042	2.008	0.574	0.227	1.830	0.714	0.192
Beta	0.331	0.339	0.343	0.332	0.338	0.342	0.013	0.011	0.011	0.014	0.009	0.009
K	4.798	9.381	13.689	4.799	9.210	13.855	1.788	1.955	2.172	1.859	2.052	2.028
Corr-coef	0.988	0.989	0.981	0.985	0.992	0.985	0.018	0.016	0.022	0.023	0.011	0.015
Eccentricity	0.964	0.964	0.968	0.968	0.969	0.972	0.011	0.013	0.014	0.008	0.008	0.008
Major axis (cm)	7.723	7.704	7.690	7.755	7.621	7.559	0.748	0.556	0.797	0.897	0.588	0.657
Minor axis (cm)	2.023	2.008	1.858	1.932	1.876	1.750	0.287	0.301	0.344	0.283	0.247	0.265
Aspect ratio	0.263	0.262	0.244	0.250	0.247	0.232	0.037	0.045	0.052	0.029	0.031	0.034
Perimeter (cm)	33.318	33.225	32.906	33.264	32.647	32.186	3.100	2.174	3.099	3.791	2.451	2.720
Relative size	0.971	0.968	0.959	0.969	0.951	0.938	0.090	0.063	0.090	0.110	0.071	0.079

**Figure 2:**
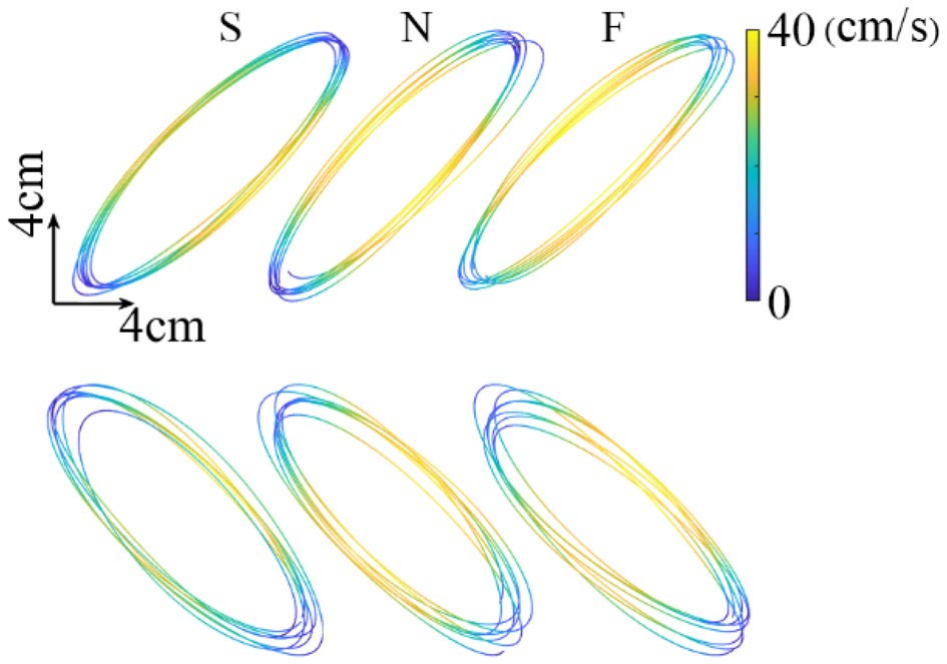
Typical drawing movements performed with the Dominant (right, upper part) and the non-dominant (left, lower parts) hands and at the three speeds (S, slow; N, normal; F, fast speed). The color scale and the color of the traces refer to the drawing velocity.

Subjects efficiently performed the task of drawing ellipses and were able to reproduce the template in a total of 6 different conditions (Fig. 2). These conditions were determined by two experimental factors: drawing speed (three levels: slow, normal, fast) and performing hand (two levels: dominant and non-dominant). An initial visible inspection of the ellipses drawn at the three tested speeds with D and ND hand, confirmed a priori simplicity of the task performed. Figure 2 shows the drawing traced by one typical subject and the modulation of the pencil velocity along the elliptical movement (yellow and blue for fast and slow speed, respectively). In the first step of data analysis, all parameters were analyzed using factorial statistics to evaluate the effects of speed and hand. In the second step, the consistency and precision of parameters were evaluated using the correlation between D and ND hands.

### Effect of speed and manual dominance on movement duration (MD)

The ART ANOVA showed that there was no statistically significant interaction between the effects of hand and speed on movement duration (MD), *F *(2, 195) = 0.01, *p* = 0.935. As expected, MD taken to draw one ellipse significantly changed when subjects were asked to perform the elliptical movement at slow and fast speeds (duration, F(2, 195) = 741.16, p < 0.0001), and the strength of the effect of seed on MD was 0.84 (η_p_^2^). It confirms that the 40 subjects were able to significantly and consistently change their average velocity when required. In favor to this, MD decreased by 34% at fast speed and increased by 112% at the slow speed relative to the reference pace (see Table 1).

**Figure 3:**
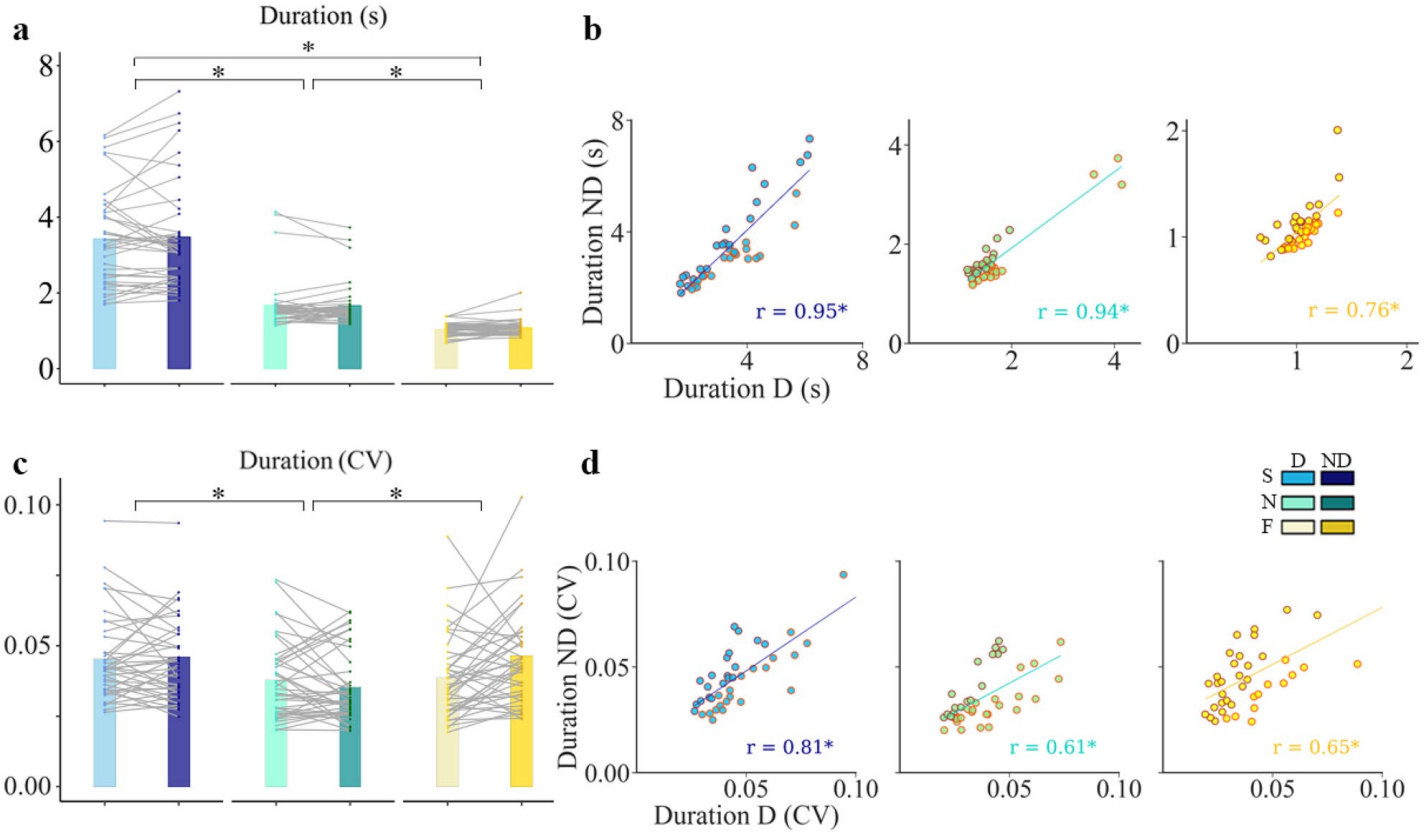
Effect of hand and speed on movement duration (MD). (**a**) Mean of MD for different conditions; insert gives the color code of each condition: S for Slow in blue; N for Normal in green; F for Fast in yellow; D for dominant hand in light colors; ND non-dominant hand in dark colors. The slope of the grey trend lines indicates individual changes of MD in the ND relative to the D hand. (**b**) Scatter plots of individual MD, each dots representing one subject (n = 40); regression lines show the correlation across the two hands (D versus ND) and for the three speeds for all subjects. Light red edge color indicates longer MD for the D hand, while dark red edge color indicates longer MD for the ND hand. (**c**) Individual and mean intra-trial variability (coefficient of variation: CV) of MD for the D and the ND hand, at the three speeds. (**d**) Scatter plots of individual CV magnitude of MD showing a strong correlation across the two hands, regardless of movement speed. Light red edge color indicates higher intra-trial variability for the D hand, while dark red edge color indicates higher intra-trial variability for the ND hand. Regression lines and r values appear superimposed on each plot. Asterisk indicates significant differences (p < 0.05).

There was a considerable inter-subject variability in MD that ranged from 1.4 to 10.96 s at slow; 0.89 to 3.99 s at normal; and 0.62 to 2.14 s at fast speed. MD was not affected by which hand was used (F(1, 195) = 0.01, p = 0.91, Fig. 3a).

Correlation accuracy (see methods: motor variability) of MD across the two hands, that is, the ability to reproduce the same duration with two hands, was estimated by computing the correlation between D and ND hands (Fig. 3b). It shows that subjects’ ND hand MD is strongly predicted by MD performed with D hand recorded during the three speed conditions. To statistically support our results, the Pearson correlation test confirmed the strong correlation of individual MD between D and ND hand for MD (r = 0.95, p < 0.001 at slow, r = 0.94, p < 0.001 at normal and r = 0.76, p < 0.001 at fast speed).

Inspection of intra-trial variability revealed a strong consistency of MD (coefficient of variation: CV < 0.05) in the reference condition (movement at the spontaneous pace with the D hand, Fig. 3c). The analysis showed that there is no significant interaction effect of hand and speed on CV of MD (F(2, 195) = 2.65, p = 0.07). However, consistency in MD along repetitions was slightly affected when subjects were asked to draw at unusual movement speeds, CV values remaining, however, less than 0.1 at fast and slow speeds (F(2, 195) = 16.02, p < 0.001, η_p_^2^ = 0.15). Nevertheless, the consistency of MD across repetition was high regardless of which hand was used (F(1, 195) = 1.98, p = 0.16).

Individual analysis (Fig. 3d) indicates that subjective intra-trial variability of the D hand predicted the ND intra-trial hand response. The correlation was always above 0.5 for the three speeds. Therefore, a subject with greater MD variability in the D hand showed greater MD variability in the ND movement (r = 0.805; r = 0.611; r = 0.649 for Slow, Normal, and Fast speed, respectively, p < 0.001).

### Effect of speed and manual dominance on Max velocity (MaxV, vigor)

The ART ANOVA analysis showed that there is no significant interaction effect of hand and speed on maxV (F(2, 195) = 0.037, p = 0.96). The result showed that speed had a significant effect on the maximum velocity (maxV, F(2,195) = 953.23, p < 0.0001, η_p_^2^ = 0.91, mean V, F(2, 195) = 752.99, p < 0.0001, η_p_^2^ = 0.89). MaxV increased by 50% and decreased by 49% from natural (19 cm/s; 0.99 Hz) speed to fast (29 cm/s; 1.51 Hz) and slow speed (10 cm/s; 0.49 Hz) conditions respectively and for the two hands. Comparing the maxV of D and ND hand indicates that it was not affected by which hand was used (F(1, 195) = 0.03, p = 0.863).

**Figure 4:**
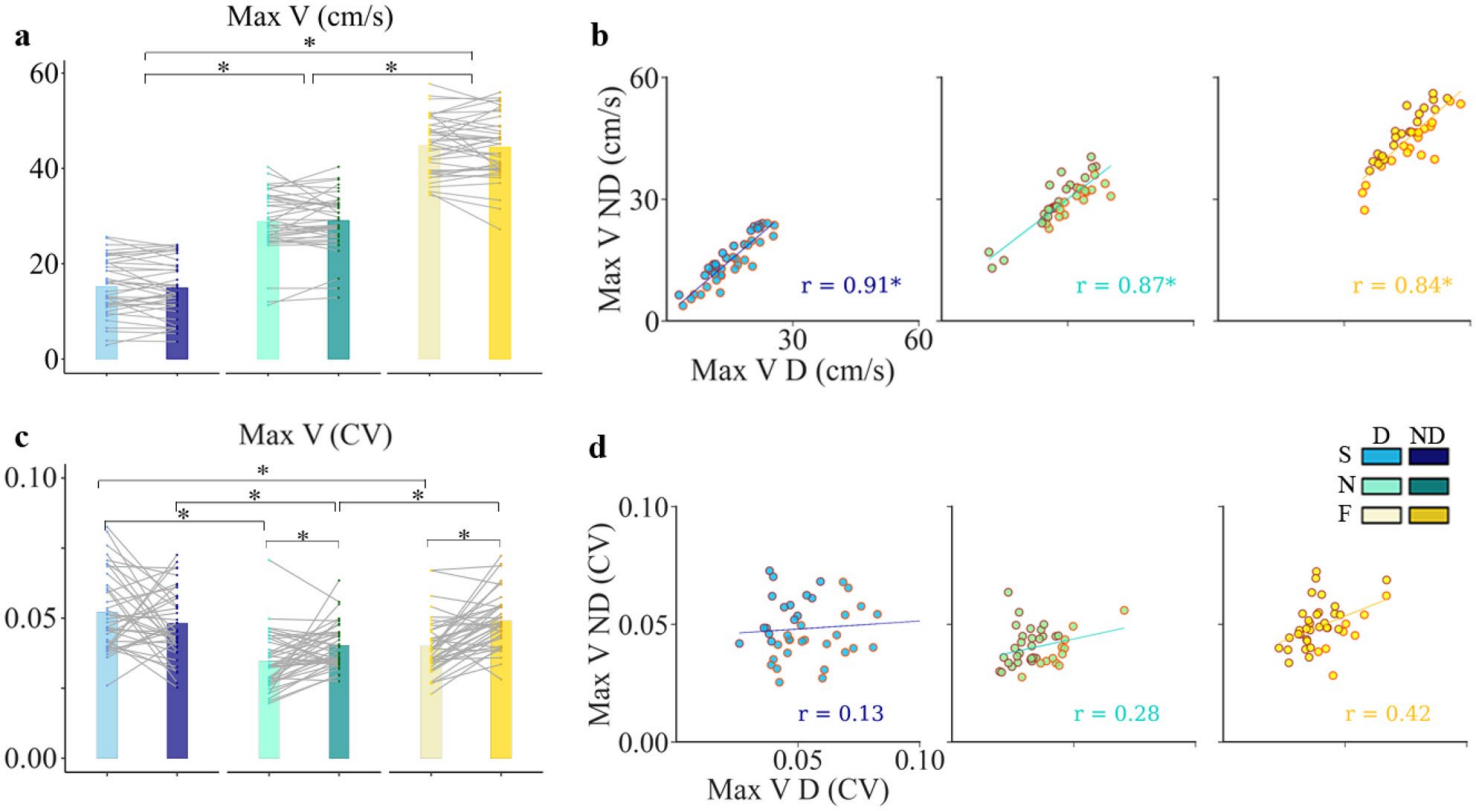
Effect of hand and speed on maximum velocity (Max V). (**a**) Mean and SD of Max V for different conditions (same color code as the previous figure). The slope of the grey trend lines indicates individual changes of Max V in the non-dominant (ND) relative to the dominant (D) hand. (**b**) Scatter plots of individual magnitude of Max V with the regression lines showing the strong correlation of individual velocity peak across the two hands and the three speeds*, *each point representing one subject (n = 40)*.* (**c**) Individual and mean intra-trial variability (CV) of Max V for the D and the ND hand, at the three speeds. (**d**) Scatter plots of individual CV magnitude of Max V showing the lack of significant correlation between hands in three speeds. Regression lines and r values appear superimposed on each plot. Asterisk indicates significant differences (p < 0.05).

As for MD, there was an important inter-subject variability (mean individual values of maxV ranging from 11 to 40 cm/s in reference condition, Fig. 4b). However, we found high correlation accuracy in maxV considering the strong correlation across the two hands, and the Pearson correlation test showed that among the 40 subjects, the correlation between maxV of D and ND hand was significant (r = 0.91, r = 0.87, r = 0.84 for slow, normal and fast speed, respectively, p < 0.001).

The results also showed a strong consistency of maxV along successive ellipses drawn with the D hand and at natural speed (CV < 0.04). ART ANOVA was applied to examine the effects of speed and hand on maxV intra-trial variability. The interaction effects of hand and speed on CV of Max V was statistically significant (F(2, 195) = 8.37, p = 0.0003, η_p_^2^ = 0.08), although this effect was weak given the small value of eta-square. Analyzing the simple main effect indicated that hand had a significant effect at normal and fast speeds (p < 0.05), while, ND and D CV of Max V were not different at slow speed. The results also showed for ND hand, CV of Max V significantly increased either when speed increased or decreased from nomal speed (p < 0.05). However, for D hand, only at slow speed CV was significantly higher than CV at normal speed. In other words, the effects of speed on intra-trial variability of max V while performing with D hand was significant only at slow speed (p < 0.05), and the difference between variability at normal and fast speed were not significant (p > 0.05). Figure 4d depicts the correlation between intra-trial variability recorded when drawing with the D and ND hands. Remarkably, in contrast to normal and fast speed, at slow speed, the intra-trial variability of maxV decreased when ellipses were drawn with the ND hand compared to the D hand (60% of the subjects reached a smaller variability at slow speed with the ND hand; refer to the light blue dots under the diagonal on Fig. 4d, left scatter plot). The correlation test showed the MaxV correlation precision was not significant at the three speed level (r = 0.13, r = 0.28, r = 0.42 for slow, normal, and fast speed, respectively). In other words, the CV value of MaxV recorded on the D side did not predict the CV value recorded on the ND hand. Figure 4d depicts that there is a tendency to increase the correlation between D and ND hands while the speed increases. However, the results of Steiger test indicated that the difference in precision correlation between three levels of speeds were not significant (p > 0.05).

### Effect of speed and manual dominance on geometrical parameters

**Figure 5:**
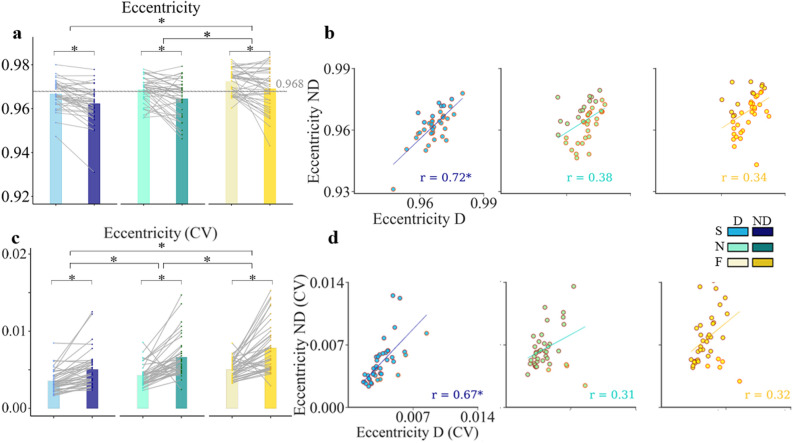
Effect of hand and speed on ellipse eccentricity*.* (**a**) Mean and SD of eccentricity for the two hands (dominant: D and non-dominant: ND) and the three speeds (S, N, F; Same abbreviations and color code as previous figures). The grey dash line shows the template eccentricity. (**b**) Scatter plots of individual magnitude of eccentricity with the regression lines showing the correlation between hands. One dot corresponds to one subject (n = 40). (**c**) Individual and mean intra-trial variability (coefficient of variation: CV) of eccentricity for the D and the ND hand, at the three speeds. Significant correlations are marked with asterisks. (**d**) Scatter plots of individual CV magnitude of eccentricity showing the lack of significant correlation across the two hands, except at slow speed. The regression lines with r values are indicated.

The mean value of eccentricity over all subjects for the three speeds tested and both hands are shown in Fig. 5a. according to the mean eccentricity values presented in Table 1, in the reference condition (natural pace and D hand); participants produced ellipses with geometrical characteristics close to the template (e.g. record mean eccentricity = 0.969; template value = 0.968).

The results indicated that the interaction of hand and speed effect on eccentricity was not statistically significant (F(2, 195) = 0.31, p = 0.74). Analyzing main effects showed that hand significantly affected ellipse eccentricity (F(1, 195) = 25.81, p < 0.0001, η_p_^2^ = 0.12) that decreased when drawing with the ND hand, thereby, the drawing pathway becoming more circular.

We found a speed-related main effect on eccentricity (F(2, 195) = 28.4, p < 0.0001, η_p_^2^ = 0.22). Precisely, Tukey post hoc analysis showed that at slow speed, hand ellipses path tended to follow a more circular path as compared to spontaneous speed (less eccentricity, p < 0.0001), whilst the ellipse’s path elongated as the drawing speed increased (higher eccentricity, p < 0.0001).

The Pearson correlation test showed that among the 40 subjects, the correlation across the two hands at normal and fast speed were insignificant (r = 0.38, r = 0.34 for normal and fast speed, respectively). In contrast, at slow speed, the D side eccentricity predicted the ND side eccentricity (r = 0.72, p < 0.001, Fig. 5b). According to the results of Steiger test, correlation accuracy was significantly higher at slow as compared to normal and fast speeds (p = 0.009).

Analysis of intra-trial consistency confirmed that the interaction effect of speed and hand on variability of eccentricity did not reach the significance level (F(1, 195) = 2.77, p = 0.065). The results of ART ANOVA indicated the presence of significant effect of hand on geometric parameters: when drawing with ND hand, variability increased compared to D side (F(1, 195) = 96.99, p < 0.0001, η_p_^2^ = 0.34, Fig. 5c). The ART ANOVA analysis also showed a main effect of speed on the CV of eccentricity (F(2, 195) = 37.9, p < 0.0001, η_p_^2^ = 0.29): at fast speed; the consistency of the drawing movement deteriorated while slow speed improved the regularity of shape produced. Similarly to the previous analysis on eccentricity correlation accuracy (higher correlation between the mean value of ND and D hands), the Steiger test showed that the subjects had more correlation precision at slow speed compared to normal and fast speed (r = 0.667, p < 0.001 for slow, r = 0.31, r = 0.32 for normal and fast speed respectively, p > 0.05).

### Effect of speed and manual dominance on the relationship between geometry and kinematics

**Figure 6:**
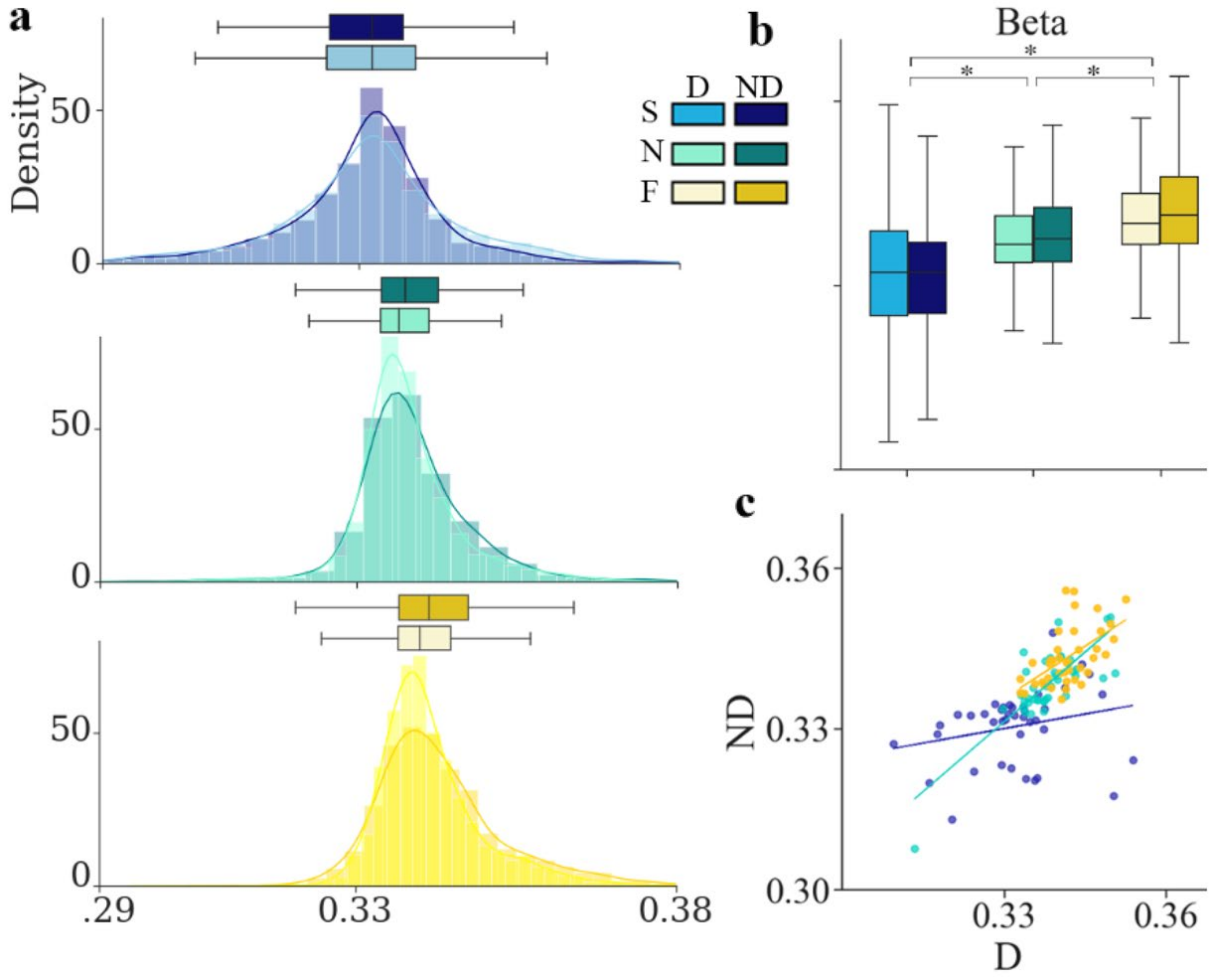
Effect of hand and speed on the Beta exponent. (**a**) Histogram showing the density distribution and the mean value with SD (upper box plots) of Beta for the two hands and the three tested speeds (same colors code as previous figures). (**b**) Boxplots of mean Beta value for the two hands and the three speeds showing lower Beta with a broader SD (0.304 to 0.36) at slow speed and higher Beta value at fast speed relative to reference condition (spontaneous speed and dominant (D) hand, green center boxplot). (**c**) Scatterplots of the correlation of Beta between D and non-dominant (ND) hand at spontaneous (green, r = 0.796), slow (blue, r = 0.238), and fast speed (yellow, r = 0.559). One dot corresponds to one subject (n = 40). Asterisks indicate significant differences (p < 0.05).

In the reference condition, the mean value of Beta exponent was 0.338, close to 0.333, which was predicted by the 1/3 power law and similar to those reported previously with adults performing similar elliptical drawing tasks (Beta = 0.339 in^20^). Values recorded from 40 subjects ranged from 0.33 to 0.351 in the reference condition. Figure 6b shows the mean values of Beta for the three speeds and the two hands.

The results of statistical analysis did not show significant interaction effect of speed and hand on beta value (F(1, 195) = 0.78, p = 0.46). We also did not find significant effect of hand on Beta (F(1, 195) = 0.06, p = 0.80), in contrast to the significant effect of speed (F(2, 195) = 106.32, p < 0.0001, η_p_^2^ = 0.52). Tukey post hoc showed that relative to reference condition, Beta significantly increased and decreased at fast and slow (p < 0.0001) speed, respectively, in the same proportion for the two-hand sides (0.332, 0.338, and 0.342 at slow, normal and fast speed respectively with D hand).

**Figure 7:**
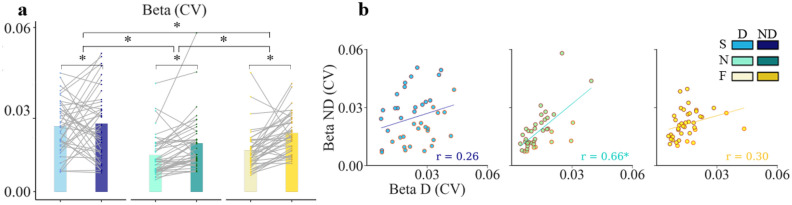
Effect of hand and speed on intra-trial variability of Beta. (**a**) Individual and mean intra-trial variability (*coefficient of variation*: CV) of Beta exponent for the D and the ND hand, at the three speeds (same colors code as previous figures); the slope of the grey trend lines indicate individual changes of CV value in the non-dominant (ND) relative to the dominant (D) hand. Significant correlations are marked with asterisks. (**b**) Scatter plots of individual CV magnitude of Beta showing the lack of significant correlation between the two hands, excepted at spontaneous speed. Each data point represents the CV of Beta of a single subject (n = 40). Light colors indicate higher intra-trial variability for the D side, while dark colors indicate higher intra-trial variability for the ND side. The regression lines with r values are indicated.

We also explored the stability issue by analyzing the Beta exponent’s CV. The ART ANOVA showed no interaction effect of hand and speed (F(1, 195) = 2.97, p = 0.054), while indicated a significant main effect of speed (F(2, 195) = 27.35, p < 0.0001, η_p_^2^ = 0.23) and hand (F(1, 195) = 4.63, p = 0.033, η_p_^2^ = 0.027). When drawing at normal pace with ND hand, the variability of the exponent was more than twice as variable as with the D hand. The lack of correlation between hands at different speeds (Fig. 7b) confirms the apparent effect of both hands and speed on Beta stability during successive drawing movements. If at spontaneous speed, individual intra-trial variability on D predicted ND hand intra-trial variability (r = 0.65), the correlation between D and ND values was, however, always less than 0.5 at slow and fast speeds. The results of Steige test showed that the differences between correlation precision at normal, slow and fast speeds were statistically significant (p < 0.05).

## Discussion

This study is characterized by two key findings: (a) Movement duration, Maximum velocity, and Speed-curvature covariation (1/3 PL) are limb-independent while geometrical parameters (i.e. primarily movement eccentricity) are systematically affected by the hand used; (b) intra-trial analyses performed over the successive drawing movements reveals a significant effect of the hand on the variability of the velocity-curvature relationship and maximum velocity. The following section discusses these differentiated modulations of motor components when drawing at different speeds with respect to the non-dominant compared to the dominant hand and further comparing the three hypothetical functional diagrams proposed in the introduction (Fig. 1).

### Effect of hand and speed on geometric parameters

The results show that manual dominance significantly influences the hand drawing pattern, with geometric parameters predominantly predicting the used hand (D or ND). When drawing with ND hand, the ellipse rounded following a more circular path with respect to the template, and the motor response along the successive movements was systematically more variable (see Figs. 6 and 7).

This modification could reflect an adaptation process to the unusual condition of drawing execution. Indeed, a less elongated path limits the sharp transition from large to small radius of curvature, which could compensate for poor control of the ND hand. This possibility agrees with previous results showing that reaching movements of the ND arm, unlike the D arm, are characterized by a strategy that does not take advantage of non-muscular forces (e.g. Coriolis or inertial forces^21^). Temporal constraints also affect the geometry of the drawing pattern. At slow speed, ellipses became less eccentric, while fast elliptical movements depicted higher eccentricity. This observation is consistent with another investigation showing more eccentric ellipses at analogous rapid movement speed^22^.

The significant effect of the hand used on geometrical parameters rules out the hypothesis of limb-independent responses. This result,a priori, agrees with the diagram C (Fig. 1c) and a control of the shape of the trajectory by motor commands generated from different modules. However, while slow speed affected the correlation precision of all other parameters (movement duration, velocity, and Beta exponent), it improved the regularity of the drawing shape (i.e. CV of eccentricity and perimeter values). Visual feedback, mainly available at slow speed, could explain the improvement in drawing accuracy. Furthermore, a longer movement duration could facilitate the interhemispheric transfer of the pre-motor output from D to ND limb. The positive effect of slow speed on intra-trial variability of hand path (Fig. 7a) accords quite well with lateralized motor control in the dominant hemisphere. Consequently, in congruence to diagram B (Fig. 1b), the visual feedback error could adjust ellipse eccentricity, a regulation facilitated at slow speed. When drawing with the ND hand, the motor command elaborated in the D hemisphere and could be transmitted via the callosal pathways to the functional module in the ND hemisphere; again, a signal flow facilitated at slow speed.

### Movement duration

The MD was not affected by hand, resulting in broad agreement with the isochrony principle^7^ (the tendency to keep the same movement duration for different movement lengths), and consistent with the idea that temporal representation is robust to new sensorimotor contexts. Remarkably, MD strongly correlated across the two hands (see Figs. 3 and 5), suggesting a shared neural substrate driving this parameter on the D and ND hands. The independence of the MD from the effector and its velocity reinforces the assumption of symmetrical blocks embodied in diagram A, assumed to be unaffected by local dynamic changes (e.g. joint torque interaction) at slow and fast speeds. The remarkable consistency of the temporal component across hands and rates could reflect the activity of a common module originating from the integration of salient cyclical input of the task, i.e. proprioceptive feedback due to abrupt changes of movement orientation^6,23^. The functional module would provide the drawing temporal structure (the beating) on the two homologous muscle groups^24^. The lack of manual dominance effect on MD is also consistent with the better temporal stability of cyclic motion recorded during bilateral coordination performed on homologous muscles compared to tasks involving non-homologous muscles^25^.

According to diagram A in Fig. 1, the symmetrical modules would generate the temporal structure of the elliptical drawing task ipsilaterally. A discrete control process would help produce the start of the first cycle and complete the hand movement at the desired position. In addition, a central pattern generator (CPG), as a low-dimensional collective variable, could pace the drawing movement on each module, regardless of the hemisphere in which it resides. Temporal tuning of cyclical hand movement might be encoded in the brainstem and spinal cord, specifically, supporting the generation of coordinated, rhythmic movement^26^. A study demonstrating that cyclical movement is less affected than discrete goal-oriented movement in stroke patients supports this possibility^27^. A time-keeping system with a sensitivity below the critical frequency of 3 Hz could also regulate rhythmic hand movement via a "timer" function presumably localized in the basal ganglia or the cerebellum. However, a non-lateralized organization is in divergence with a previous proposal of a rhythmical control of bilateral hand movement located in the contralateral hemisphere of the D hand^28^. This apparent discrepancy could stem from the task tested. In contrast to an unimanual task, a synchronous bimanual task requires postural adjustment anticipating the mechanical effect of one hemibody on the other one and motor commands relying on asymmetric neural substrates.

When subjects were asked to draw at fast or slow speeds, intra-trial MD variability remained low, indicating that hand velocities can be modulated along with the same movement amplitude. According to the isochrony principle, one might have predicted a shorter or a more extended perimeter at slow and fast speed, respectively, to compensate for the required drawing speed and maintain equal MD, which is an observation not verified here. In contrast, the result showing participants' ability to adopt movement duration deviating from the spontaneous duration indicates the versatility of temporal representations. Indeed, the CV of MD never exceeded 0.1 at slow and fast speeds. This observation is all the more surprising because it is well known that humans tend to avoid unnatural slow speed because movements with a longer duration are more effortful as compared to shorter movements against the same force^29^. Likewise, slow displacement requires more neural and attentional resources^30,31^.

### Movement vigor

The isochrony principle and the selection of a constant movement duration are regularly suggested as parameters explicitly controlled^12,32^. In contrast, the present capacity to accurately adopt various movement vigor, a term that refers to peak velocity (i.e. the modulation of the energy invested into voluntary movements^33^), is a relatively new observation. Upper limb movements are continuously executed during daily life at different speeds depending on intrinsic (mental state, emotion, intention, etc.) and extrinsic (e.g. postural or temporal restrictions) conditions. The amplitude of the maximum hand velocity was idiosyncratic and unaffected by the hand selection (Figs. 4 and 5), suggesting that vigor, as a component of the goal, reflects an intrinsic value guiding the execution of the task rather than its details. Indeed, in the optimal motor control and reward-oriented actions theories framework, vigor has previously been modeled as a decision-making process^34,35^. Movement vigor would thus reflect a subjective value^36^, i.e. a relatively stable and distinctive attribute of individuality. Precisely, in addition to anthropometric factors, an individual's preferred movement speed would be selected by combining several factors as the minimization of metabolic energy expenditure with individual traits (e.g. personality traits linked to time-related decision making). The present results assessing the consistency of idiosyncratic maximum velocity across various modalities agree that vigor reflects a personal feature. During ellipse drawing, the inertia of hand movement is small. Inter-subject anthropometric differences are thus negligible, at least for drawing movement performed at spontaneous speed, and cannot explain subjective hand velocities. Instead, we speculate that this parameter reflects stylistic variations during intransitive expressive acts performed with distal limbs.

According to this reasoning, the vigor could rely on an asymmetric control, as depicted in diagram B (Fig. 1), driving similar joints for both arms and preserving the subjective values of the task. Indeed, a lateralized command accords with previous proposals that personal traits and cognitive style depend upon cerebral dominance^37^, and the right hemisphere is sensitive to the emotional connotation of stimuli in the decision-making process^38^.

However, intra-trial variability of vigor was significantly affected by hand and drawing speed. The instability of individual peak velocity, ellipse after ellipse could result from the fine-tuning of muscle activity of D and ND hands to produce personal hand velocity. Each hemisphere should have the necessary information based on online proprioceptive feedback when required vigor deviates from spontaneous vigor. A lateralized control (diagram B) of the velocity peak seems thus the most appropriate solution to generate a specific muscle activity. Still, one cannot exclude symmetrical commands because the current drawing task is not a dexterity task requiring independent and fine finger displacements. Indeed, the present task mainly results from the activity of the elbow and shoulder muscles, which are primarily controlled ipsilaterally and receive bilateral inputs from many sites along the neural axis^39^.

### Consistency of 1/3 PL across effectors, speeds, and subjects

We did not find a significant effect of manual dominance on the velocity-curvature relationship, at least when ellipses were performed at spontaneous speed. This result is a priori in line with the view that the covariation, rather than being a by-product of local mechanisms^40^, originates from the motion planning stages^8^. Invariance of Beta exponent was also expected based on the visuomotor coupling and the 1/3 PL illusion^41^, that is the perception of a point-light display (i.e. a generic trajectory unattached to a specific body part) moving at constant velocity when its kinematic complies with the 1/3 PL. As a matter of fact, this illusion indirectly shows a top-down influence of the curvature-velocity covariation on visual perception, a hierarchical organization embodied by diagram A (Fig. 1).

However, the ellipse-by-ellipse analysis reveals variability in the Beta exponent (i.e. the straight-line slope on a log-speed log-curvature scale). Slow and fast speeds significantly affected Beta’s correlation accuracy and precision, an observation not visible in previous investigations based on averaged values across a limited number of repetitions. In fact, manual dominance predicts intertrial variability and Beta CV value that increased when drawing with the ND compared to the D hand. Generally, motor variability is interpreted as an unwanted by-product of a noisy nervous system^32,42^. Another assumption is that variability could signal implicit exploration where the nervous system intentionally varies the motor commands searching for actions yielding the most significant success^43^. Thus, the unsteady Beta value recorded at slow and fast speeds and drawing by ND hand could reflect sensorimotor adaptation to unpracticed conditions. This hypothesis could be verified with a dedicated protocol, comprising the repetition of numerous trials performed with ND hand and at non-spontaneous speed. The sensitivity of Beta to both dynamic context and limb suggests that the 1/3 PL is represented with motoric reference rather than in purely abstract terms. Accordingly, although an invariant status is recurrently attributed to the Beta exponent, it is nevertheless frequently found variable and task-dependent. For instance, the Beta value depends on movement frequency in a decreasing manner^22,44^. Likewise, the exponent of the power function in water is significantly greater than in air, and the power relationship is violated when changing the dynamic context^13^. The current instability of the Beta value is thus compatible with the engagement of several neural regions assuming the covariation. For instance, it has been demonstrated that the neural circuitry carrying the imprint of the 1/3 PL is not limited to one specific locus or modality but is shared by visual, sensorimotor, motor cortical and subcortical areas^45^. The result is not surprising, considering that a covariation needs at least two components: in the case of the 1/3 PL, the curvature of a trajectory can only be identified with visual inputs.

Considering the possible distributed nature of the network dealing with the 1/3 PL, the functional module producing the speed-curvature covariation embodied in diagram B (Fig. 1) could receive the projections of multiple areas. Stored in the dominant hemisphere, it could adjust the motor output when a change in the dynamic context is required or when drawing with the non-preferred hand, from D hemisphere to ND hemisphere via callosal pathways.

In conclusion, using advanced behavioral analysis, our results indicate different modulation of movement descriptors when drawing at different speeds with the ND compared to the D hand. The specific effects of manual dominance most likely suggest that various neural strategies are used to control the task parameters, which are grouped into three categories. The first includes the duration of movement, a component independent of the limb used and without any reference to the dynamic context of the activity, possibly generated by low-level neural substrates. Vigor and beta exponent, in which variability is affected by manual dominance, belong to the second category. The third included the geometric parameters, which are limb and movement speed-dependent. In terms of neural control, this last category supposed that the descending pathways provided by each hemisphere generate separate signals on the relevant limb. In summary, the three-category scheme does not go from the most abstract component to the least abstract, as implied by the hypothesis of a top-down hierarchical organization of the motor command. Instead, it suggests a distributed network in contrast to a central executive system initiated in the frontal lobes and separated from sensorimotor control^16,46^.

## Methods

### Participants

Forty healthy subjects (14 males and 26 females), with normal or corrected-to-normal vision and naive to the task, participated in this study. None of the participants had reported neurological, psychiatric, or other relevant medical problems affecting motor performance. The Ethics Committee of the Regione Emilia Romagna approved the experimental procedures (Comitato Etico di Area Vasta Emilia Centro Della Regione Emilia-Romagna; Ref. EM255-2020 UniFe/170592 EM Estensione) and all methods were performed in accordance with the Declaration of Helsinki. Accordingly, each participant was made aware of the protocol and provided written informed consent before participating in the study.

A statistical power analysis was performed for sample size estimation, based on data from a published study by Pillipse^47^, comparing the effects of speed, submovements, joint complexity, radius of curvature on the consistency of power law. The effect size (ES) in this study ranged from0.74 to 0.88, considered to be high according to Cohen's criteria. With an alpha = 0.05, ES = 0.88 and power = 0.80, the projected sample size needed with this effect size (GPower 3.1) is approximately N = 40 for this study^48^.

In order to determine the hand Dominance we used the Edinburgh Handedness Inventory which is a scale to measure the degree of hand laterality in daily activities such as following: writing, drawing, throwing, using scissors, brushing teeth, using a knife, using a spoon, using a broom, striking a match, and opening a box. Handedness was scored numerically for each subject according to the method proposed by Oldfield (1971) to calculate a laterality quotient (LQ)^49^. The LQ ranges from -100 (totally left-handed) to + 100 (totally right-handed). The mean value of LQ for 40 subjects was 66.1 (two left-handed: − 52.8 and 38 right-handed: 79.37).

### Procedure

Under an ordinary lighting condition, we organized the whole recordings in a quiet room. The coordinates of drawing movements were recorded via a Wacom tablet (Bamboo slate; temporal resolution: 200 samples/s; resolution: 1748 by 2551; Active area: 210 × 297 mm), placed in a horizontal position in front of the sitting participants.

Subjects were required to perform a simple motor task, drawing ten ellipses continuously on a template ellipse with three different speeds and hands separately. The participants were told that these templates were just an indicator of the ellipse size and that the task was not a copying task but consisted of producing natural movements at a self-paced rate.

The template ellipse to be traced was drawn on standard-sized (A4) white sheets set on the table. The bamboo ink pen leaves a visual trace on the paper during drawing, and therefore, after 2 or 3 ellipses traced, the template was no more visible, and the possible visual online motor control was limited.

The geometrical features of the template were defined by its eccentricity of 0.968, major semi-axis of 8 cm, and minor semi-axis of 2 cm. In addition, the major axis of the ellipse template was rotated by 45° and − 45° for the right and left-hand movements, respectively.

Each participant carried out six different movement conditions considering experimental factors, the drawing speed (3 levels) and the performing hand (2 levels). A metronome indicated the rate of the movements for only spontaneous speed (referred to as normal speed in this paper) with a period of 80 beats per minute (BPM) in order to uniform the reference pace among subjects to produce slower and faster speed. The pace of 80 BPM allows subjects to easier chose their preferred slow or fast speed and avoids the ceiling effect in very slow or fast subjects. During the training with the metronome, subjects were free to choose the position along the trajectory that was phase-locked to the beats. After several cycles, when the subject memorized the pace synchronized with the metronome and was satisfied that a stable phase-locking had been attained in the training session, the metronome stopped, and the free-running performance was recorded for ten ellipses without the metronome. The training sessions with metronome were conducted once before the start of recording data (for each hand). For slow and fast speed, subjects were asked to produce their preferred slower and faster speed relative to their spontaneous speed. In order to maximize the degree of similarity of motor task between two hands (similar muscular activation), the right and left hand movement direction during drawing of the ellipse were determined counterclockwise and clockwise, respectively.

Each subject executed ten successive repetitions per condition. In the end, each subject performed a total of 60 repetitions, presented in two blocks, separated by short breaks. The order in which these repetitions were conducted was randomized with the constraint that the same movement type could not occur in the successive trial. At the beginning of the experiment, subjects were asked to synchronize their movement to the beats of a metronome and try to memorize it as the spontaneous speed.

### Measures

For each repetition, the first and two last ellipses were excluded from data analysis. Then kinematics and geometrical parameters of the seven remained traces were analyzed by offline processing of the coordinates data. The *x*, *y* position samples were low pass filtered (0.07 Hz cut-off, zero-phase-lag Butterworth filter).

### Geometrical parameters

The global geometric accuracy of the performances was measured considering several parameters: minor semi-axis of the ellipse, eccentricity (elongation of the ellipse, which is calculated by this equation e = √1–(b/a)^2^, a is major semi-axis, and b is minor semi-axis). Angle of rotation (the inclination of the major axis of the traces to the horizontal plane) and perimeter of the ellipse were also computed.

### Relation between geometry and kinematics

The relationship between the geometrical form of a movement and its velocity is well characterized by the empirical relation V(t) = KC(t)^1–β^ where: V is the angular velocity, C is the curvature, at time t; K is a constant, named the velocity gain factor, which depends on the type of movement. To compute the adequacy of the 1/3 power law, two levels of analysis are undertaken^50^. The initial level adopts a straightforward approach of analyzing each movement cycle, which its outcome are angular velocity and radius of curvature. In the second step, we calculate β exponent in equation (V(t) = KC(t)^1–β^).

*Velocity and radius of curvature:* To determine a metric relation between handwriting speed and local line curvature, which requires numerical algorithms in a discrete fashion, we use an exact relation to estimate the local curvature around a point lying on the written line. The method used is based on previous papers **(Stucchi, unpublished)** and extensively described in the *supplementary materials* (see “Appendix”).

### Motor variability

Variability in performance may stem from multiple causes (sensory, neuromuscular, or cortical) during the drawing task preparation and execution. In the present case, one could expect kinematic variability in response to unusual motor conditions to perform the task (e.g. fast and slow speeds drawing and movement performed with the ND hand). The *accuracy* of the motor response was estimated by first averaging all repeats of an ellipse on the D and ND hand and then by computing the correlation coefficient between individual values obtained for each hand. In the other words, correlation accuracy here interpreted as a index which indicates how ND hand produced similar respond relative to D hand. We also performed a trial-by-trial analysis to preserve the data for each ellipse and assess the variability of the motor response ellipse after ellipse (*motor precision*). We thus estimated the motor response's precision (inverse variance) by considering the intra-trial variability through the coefficient of variation (CV) over the seven successive ellipses. CV values were then averaged for each speed, hand, and subject and then averaged across all subjects. Finally, the correlation between individual CV obtained on the D hand and the CV obtained on the ND was calculated as an index of the motor precision of two hands.

### Statistical analyses

Statistical analyses were performed with Rstudio and SPSS software (version 28, IBM SPSS Statistics, Armonk, NY, USA) on all variables. The Aligned Rank Transform (ART) ANOVA, a robust non-parametric method for multi-factorial experiments, was performed to analyze the interaction as well as the main effects of independent variables (α = 0.05).The independent variables were speed (3 levels; slow, normal, and fast) and hand (2 levels; D and ND). When necessary, post-hoc tests were conducted with Tuckey’s test. Moreover, in order to tests the significance of the difference between two dependent correlations we used Steiger Test.

## Supplementary Information

Supplementary Information.

## Data Availability

Collected data regarding our experiment are made freely available to the research community: (https://github.com/lucaoneto/COGN2021_Elipses/tree/main/data).
