# Supplementary material for: The effect of the preferred hand on drawing movement
Source: Sci Rep. 2023 May 22;13:8264. doi: 10.1038/s41598-023-34861-x (PMC10202903; doi:10.1038/s41598-023-34861-x)
Supplement: Supplementary file 1 — Supplementary Information. [file 41598_2023_34861_MOESM1_ESM.docx]

**Appendix**

*Velocity and radius of curvature:* To determine a metric relation between handwriting speed and local line curvature, which requires numerical algorithms in a discrete fashion, we use an exact relation to estimate the local curvature around a point lying on the written line. The method requires the use of three consecutive line points, as indicated in Fig. S1. Specifically, we estimate the curvature at point 2 from the radius of the circumscribed circle to the triangle defined by the three considered points. This definition is based on the continuum analog in which the radius of curvature at a line point (here 2) is taken as the radius of the circumscribed circle in the limit that points (1 and 3) tend to point 2. According to planar geometry, the radius R of the circumscribed circle given three points of coordinates (x_i_, y_i_), i = 1, 2, 3, is obtained from the equation,

1. $R=\frac{S}{4\cos(\theta_{1}/2)\cos(\theta_{2}/2)\cos(\theta_{3}/2)}$

where S = (r_12_+r_23_+r_13_)/2 is the semi perimeter and θ_i_ are the internal angles of the inscribed triangle (Fig. S1). To implement Eq. (1) in practical calculations, it is convenient to work out a simpler algebraic expression for R. The procedure starts by finding the equation of the straight line y = a+bx going through points (1) and (3), yielding

1. $a=\frac{x_{1}y_{3}-x_{3}y_{1}}{x_{1}-x_{3}}, and b=\frac{y_{1}-y_{3}}{x_{1}-x_{3}}$

Next, one calculates the shortest distance of the point (2) to the straight line, denoted as r. The result is,

1. $\frac{\left|y_{2}-a\right.-\left.bx_{2}\right|}{\sqrt{1+b^{2}}}$

The interest about r is that it is inversely proportional to the radius of curvature R, as we will see below.


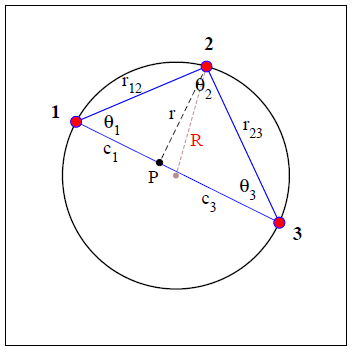


Figure S1- Geometrical construction for determining the 'local radius of curvature' R around point 2. The dots numbered (1,2,3) represent three sequentially drawn line points. The straight line joining (1 and 3) is referred to in the text as the equation y = a+bx. The distance between (1 and 2) is r12 and between (2 and 3) r_23_. The length r is the shortest distance of (2) to the straight-line y, (θ_1_, θ_2_, θ_3_) are the internal angles, and (c_1_, c_3_) two internal distances, such that c_1_ + c_3_ = r_13_, the distance between (1 and 3). The angle 1P2=π/2.

For the angles θ_1_ and θ_3_ one has cos (θ_1_) = c_1_/r_12_ and cos (θ_3_) = c_3_/r_23_, respectively, from which we obtain

1. $\begin{matrix} \cos(\theta_{1}/2)=\sqrt{\left(r_{12}+c_{1}\right)/\left(2r_{12}\right)} \\ \cos(\theta_{3}/2)=\sqrt{\left(r_{23}+c_{3}\right)/\left(2r_{23}\right)} \end{matrix}$

Regarding θ_2_, we use cos (θ_2_/2) = sin (θ_1_/2 + θ_3_/2) together with the expressions in Eq. (4) we can write,

1. $\cos(\theta_{2}/2)=\frac{1}{2\sqrt{r_{12}r_{23}}}\left(\sqrt{\left(r_{12}-c_{1}\right)\left(r_{23}+c_{3}\right)}\right)+\left(\sqrt{\left(r_{12}+c_{1}\right)\left(r_{23}-c_{3}\right)}\right)$

Using Eq. (4) and Eq. (5) into Eq. (1), we arrive at the final form,

1. $R=\frac{r_{12}r_{23}}{2r}$

Note that if the underlying written line would be a circle of radius R0, then r_12_ = r_23_ = √2R_0_ (assuming for simplicity that the handwriting angular speed is constant in this case) and r = R0, then one finds R = R_0_. Finally, the handwriting speed at (2) can be estimated as the mean speed in going from point (1) to (2) and from (2) to (3), i.e.

1. $v(2)=\frac{1}{2}\left(v_{12}+v_{23}\right)=\frac{1}{2}\left(\frac{r_{12}}{t}+\frac{r_{23}}{t}\right)$

*β exponent and velocity gain factor in Two-third power law:* The calculation of power law may be implemented by using a linear regression analysis of log *V(t)* on log R(t) based upon Equation 3. The slope and the intercept parameter of the log V-log R linear regression can be used to estimate the exponent β and the velocity gain factor K of the power law, respectively.

The data used for the regression should conform to the limits of Equation 2; hence, low curvature sections should be excluded. In this case, all radius values three times greater than the drawing board were rejected.
